# Supplementary material for: Enhancing domain generalization in the AI-based analysis of chest radiographs with federated learning
Source: Sci Rep. 2023 Dec 19;13:22576. doi: 10.1038/s41598-023-49956-8 (PMC10730705; doi:10.1038/s41598-023-49956-8)
Supplement: Supplementary file 1 — Supplementary Tables. [file 41598_2023_49956_MOESM1_ESM.pdf]

Supplementary Information

Table S1: On-Domain Evaluation. The results are represented as the accuracy, sensitivity, and specificity percentage values averaged over all imaging findings, i.e., cardiomegaly, pleural effusion, pneumonia, atelectasis, consolidation, and pneumothorax as well as no abnormality for each dataset, utilizing the ResNet50 architecture (as the prototypical Convolutional Neural Network) and the ViT architecture (as the prototypical Transformer Network). Two training strategies were used, i.e., local training and collaborative training (i.e., federated learning). The datasets employed in this study were the VinDr-CXR, ChestX-ray14, CheXpert, MIMIC-CXR, and PadChest datasets with n=15,000, n=86,524, n=128,356, n=170,153, and n=88,480 training radiographs, and n=3,000, n=25,596, n=39,824, n=43,768, and n=22,045 test radiographs.

Test Dataset	Training Strategy	Convolutional Neural Network			Transformer Network		
		Accuracy	Sensitivity	Specificity	Accuracy	Sensitivity	Specificity
**VinDr-CXR**	**Local**	84.9 ± 3.9	80.9 ± 8.7	83.8 ± 6.1	84.7 ± 7.2	84.8 ± 8.9	84.4 ± 7.5
	**Collaborative**	88.4 ± 5.2	88.3 ± 5.9	87.4 ± 6.1	89.1 ± 4.9	89.0 ± 5.2	88.8 ± 5.3
**ChestX-ray14**	**Local**	70.7 ± 6.4	70.7 ± 9.8	71.8 ± 7.5	70.2 ± 7.5	72.0 ± 9.3	71.0 ± 8.8
	**Collaborative**	71.6 ± 7.8	74.1 ± 8.7	72.4 ± 8.8	72.3 ± 6.3	73.1 ± 9.6	73.4 ± 7.4
**CheXpert**	**Local**	71.2 ± 9.4	78.3 ± 5.7	69.9 ± 10.3	73.4 ± 8.2	76.7 ± 7.7	72.5 ± 8.5
	**Collaborative**	73.5 ± 10.5	75.7 ± 9.6	72.5 ± 11.9	74.6 ± 7.7	75.4 ± 8.0	74.1 ± 8.0
**MIMIC-CXR**	**Local**	74.0 ± 5.6	77.9 ± 7.0	73.2 ± 6.3	74.0 ± 5.7	78.9 ± 6.7	72.9 ± 6.5
	**Collaborative**	74.4 ± 5.6	77.7 ± 6.4	73.6 ± 5.9	73.5 ± 6.6	78.3 ± 6.3	72.8 ± 7.3
**PadChest**	**Local**	80.8 ± 4.4	83.0 ± 6.0	80.3 ± 5.0	78.5 ± 5.7	83.2 ± 6.7	78.0 ± 6.1
	**Collaborative**	80.8 ± 5.5	84.4 ± 5.3	80.3 ± 6.1	81.6 ± 5.2	84.5 ± 6.0	81.0 ± 6.0

Table S2: Off-domain Evaluation of Performance of the Convolutional Neural Network – Standardized Training Data Sizes. Data are accuracy, sensitivity, and specificity, averaged over all imaging findings when trained locally or collaboratively (i.e., utilizing federated learning) and tested on another dataset. The collaborative training strategy used the remaining four datasets, each contributing n=15,000 training radiographs. Notably, the VinDr-CXR local model was trained using all available radiographs (*), i.e., n=15,000, while the local models of the other datasets were trained using n=60,000 radiographs. The test sets included n=3,000 (VinDr-CXR dataset), n=25,596 (ChestX-ray14 dataset), n=39,824 (CheXpert dataset), n=43,768 (MIMIC-CXR dataset), and n=22,045 (PadChest dataset) radiographs, respectively. OND: On-Domain.

**Train on:**		**Evaluation Metric**	**Test on:**				
**Training Strategy**	**Dataset [Size]**		**VinDr-CXR**	**ChestX-ray14**	**CheXpert**	**MIMIC-CXR**	**PadChest**
**Local Training**	**VinDr-CXR [n=15000] (*)**	**Accuracy**	OND	54.3 ± 10.4	64.0 ± 12.6	63.0 ± 6.7	71.3 ± 7.3
		**Sensitivity**		68.9 ± 11.4	65.4 ± 16.9	71.4 ± 9.3	71.0 ± 12.2
		**Specificity**		53.6 ± 14.6	62.5 ± 14.8	61.5 ± 9.0	70.6 ± 7.7
	**ChestX-ray14 [n=60000]**	**Accuracy**	79.2 ± 7.7	OND	65.0 ± 10.8	67.1 ± 7.1	75.2 ± 7.6
		**Sensitivity**	76.8 ± 8.0		72.9 ± 7.3	71.9 ± 9.1	74.1 ± 10.3
		**Specificity**	79.4 ± 8.0		63.7 ± 12.2	66.0 ± 7.6	74.5 ± 8.3
	**CheXpert [n=60000]**	**Accuracy**	79.1 ± 9.8	66.6 ± 7.1	OND	71.1 ± 6.1	76.4 ± 7.2
		**Sensitivity**	78.5 ± 9.8	69.8 ± 9.2		71.4 ± 11.4	74.1 ± 10.6
		**Specificity**	78.6 ± 10.3	67.3 ± 9.2		70.7 ± 6.9	76.2 ± 7.4
	**MIMIC-CXR [n=60000]**	**Accuracy**	81.3 ± 6.7	67.6 ± 8.2	68.3 ± 9.8	OND	74.4 ± 8.4
		**Sensitivity**	78.8 ± 9.2	67.8 ± 9.7	74.0 ± 6.5		78.3 ± 8.7
		**Specificity**	80.5 ± 8.1	68.3 ± 9.8	67.4 ± 10.8		73.5 ± 9.1
	**PadChest [n=60000]**	**Accuracy**	77.9 ± 9.9	62.9 ± 10.6	68.5 ± 9.7	66.8 ± 7.2	OND
		**Sensitivity**	77.9 ± 8.9	68.8 ± 9.8	68.2 ± 12.7	72.1 ± 9.3	
		**Specificity**	77.3 ± 10.3	63.2 ± 12.6	67.5 ± 10.6	65.6 ± 8.2	
**Collaborative Training**	**All Datasets [n=4 x 15000]**	**Accuracy**	82.5 ± 6.5	65.9 ± 9.0	69.1 ± 10.0	67.1 ± 6.8	73.3 ± 9.1
		**Sensitivity**	77.0 ± 9.3	70.3 ± 8.2	70.2 ± 11.5	75.2 ± 6.4	79.2 ± 9.4
		**Specificity**	82.5 ± 6.8	66.4 ± 10.7	68.0 ± 11.2	65.8 ± 7.3	72.8 ± 9.1

Table S3: Off-domain Evaluation of Performance of the Vision Transformer – Standardized Training Data Sizes. Data organization as in **Table S2**.

**Train on:**		**Evaluation Metric**	**Test on:**				
**Training Strategy**	**Dataset [Size]**		**VinDr-CXR**	**ChestX-ray14**	**CheXpert**	**MIMIC-CXR**	**PadChest**
**Local Training**	**VinDr-CXR [n=15000] (*)**	**Accuracy**	OND	54.0 ± 11.7	64.1 ± 13.8	63.4 ± 7.2	69.5 ± 9.7
		**Sensitivity**		72.8 ± 12.2	67.9 ± 17.4	75.0 ± 7.6	79.2 ± 8.0
		**Specificity**		53.3 ± 16.3	62.2 ± 16.6	61.4 ± 8.7	68.7 ± 9.7
	**ChestX-ray14 [n=60000]**	**Accuracy**	79.4 ± 10.9	OND	67.7 ± 8.9	67.1 ± 6.5	74.7 ± 8.3
		**Sensitivity**	78.2 ± 9.1		71.7 ± 8.1	74.6 ± 7.5	78.0 ± 8.6
		**Specificity**	79.8 ± 11.4		66.8 ± 9.8	65.9 ± 7.2	74.1 ± 8.9
	**CheXpert [n=60000]**	**Accuracy**	82.4 ± 6.2	67.77 ± 8.6	OND	71.3 ± 6.1	75.8 ± 6.5
		**Sensitivity**	76.7 ± 13.7	71.3 ± 10.6		73.2 ± 10.2	76.8 ± 11.5
		**Specificity**	82.6 ± 6.8	68.6 ± 10.5		70.5 ± 7.2	75.3 ± 7.0
	**MIMIC-CXR [n=60000]**	**Accuracy**	84.1 ± 5.9	67.8 ± 6.4	69.6 ± 8.7	OND	77.1 ± 6.4
		**Sensitivity**	82.1 ± 9.6	69.9 ± 7.6	74.5 ± 6.3		80.1 ± 7.9
		**Specificity**	83.1 ± 7.9	68.7 ± 7.9	68.6 ± 9.6		76.5 ± 6.8
	**PadChest [n=60000]**	**Accuracy**	82.3 ± 6.3	64.0 ± 9.1	67.3 ± 10.6	67.2 ± 7.9	OND
		**Sensitivity**	82.1 ± 7.0	70.3 ± 8.8	71.2 ± 12.1	75.1 ± 9.8	
		**Specificity**	82.1 ± 6.5	64.6 ± 11.2	65.9 ± 11.7	65.8 ± 9.2	
**Collaborative Training**	**All Datasets [n=4 x 15000]**	**Accuracy**	83.5 ± 6.1	66.1 ± 8.4	69.3 ± 10.3	69.7 ± 6.1	76.5 ± 6.1
		**Sensitivity**	84.7 ± 7.7	71.5 ± 9.5	72.5 ± 10.0	75.0 ± 6.4	80.9 ± 6.8
		**Specificity**	83.2 ± 6.5	67.2 ± 10.8	68.3 ± 11.4	68.9 ± 6.8	75.9 ± 6.4
